# Bioelectromagnetic Platform for Cell, Tissue, and In Vivo Stimulation

**DOI:** 10.3390/bios11080248

**Published:** 2021-07-25

**Authors:** Ryan C. Ashbaugh, Lalita Udpa, Ron R. Israeli, Assaf A. Gilad, Galit Pelled

**Affiliations:** 1 Department of Electrical and Computer Engineering, Michigan State University, East Lansing, MI 48824, USA; ashbau12@msu.edu (R.C.A.); udpal@msu.edu (L.U.); 2 Neuroengineering Division, Michigan State University, East Lansing, MI 48824, USA; israeli2@msu.edu; 3 Department of Biomedical Engineering, Michigan State University, East Lansing, MI 48824, USA; 4 Department of Radiology, Michigan State University, East Lansing, MI 48824, USA; 5 Synthetic Biology Division, Michigan State University, East Lansing, MI 48824, USA

**Keywords:** high-throughput magnetogenetics, in vivo stimulation, head-fixed experiments, neuromodulation, minimally-invasive brain stimulation

## Abstract

Magnetogenetics is a new field that utilizes electromagnetic fields to remotely control cellular activity. In addition to the development of the biological genetic tools, this approach requires designing hardware with a specific set of demands for the electromagnets used to provide the desired stimulation for electrophysiology and imaging experiments. Here, we present a universal stimulus delivery system comprising four magnet designs compatible with electrophysiology, fluorescence and luminescence imaging, microscopy, and freely behaving animal experiments. The overall system includes a low-cost stimulation controller that enables rapid switching between active and sham stimulation trials as well as precise control of stimulation delivery thereby enabling repeatable and reproducible measurements.

## 1. Introduction

The rapid growth of research interest in magnetogenetics in the past decade has resulted in a broad range of bioelectromagnetic stimulation applications [1], creating a demand for sophisticated stimulus delivery systems. Many biological systems can be magnetically stimulated to regulate gene expression or neural activity, and stimulation parameters can vary significantly depending on the mechanisms employed to elicit responses [1]. In contrast to visible light, low frequency and DC magnetic fields easily penetrate soft tissue and bone, potentially allowing for minimally invasive and wireless stimulation. High costs of bioelectromagnetic stimulation devices and a lack of systematic analysis of electromagnetic stimulus fields serve as a barrier to designing quantitative studies and replicating results in magnetogenetics experiments. Development of magnetic sensitive pathways, like those using nanoparticles [2] and proteins like the electromagnetic perceptive gene (EPG) [3,4,5,6,7], have contributed to making magnetic stimulus delivery for wide ranging applications increasingly important. Furthermore, recent studies which show that humans may also have magnetoperception [8] serve to increase the demand for easy to implement and versatile electromagnetic stimulation devices.

One solution, which is well suited for stimulating multi-well plates, includes the coil array of [9], which consists of an array of coils positioned to fit underneath each well of a 24-well plate. The coils are then used to deliver a pulsed time varying electromagnetic field stimulus having magnetic flux densities in the range of 1.0 to 1.2 mT.

Another solution for magnetic stimulus delivery includes the use of an induction heater [2]; however, these are limited in their ability to be integrated into a wide variety of experimental protocols. Therefore, it is beneficial to design and build electromagnets which can be more easily incorporated into a wide variety of applications. Custom stimulation coils demonstrate improved integration into microscopy applications [10,11], and we aim to build on this flexibility and emphasize detailed stimulation validation. Furthermore, repeatability, uniformity, a negative control condition, and ease of use are critical properties of interest in magnetic stimulus systems.

Here, we present work conducted toward developing a magnetogenetics bioelectromagnet stimulation platform which is low cost, versatile, easy to use, and affords a high degree of control over stimulation parameters. The electromagnet designs presented in this paper are applicable for electrophysiology, microscopy, fluorescence and luminescence imaging, and also to stimulate in freely behaving animals.

We developed four designs, where each design is unique to accommodate application specific physical constraints as well as maintain uniformity in the target area. Double wrapping coils as described in Kirschvink et al. [12] allows experiments to be tested with a negative control. Additionally, a low-cost stimulation controller along with an accompanying graphical interface provides a user-friendly way to switch between active and sham stimulation conditions and reproduce specific stimulus parameters.

## 2. Materials and Methods

### 2.1. Applications

The primary use of the electromagnet systems we designed are microscopy, luminescence, and fluorescence imaging, in vivo electrophysiology, and freely behaving animal experiments. For each application, our goal was to design an electromagnet that can deliver the desired magnetic flux given various constraints including power consumption, coil and sample temperature, and coil size. Evidence suggests that applying magnetic flux densities >50 mT [3,7,13] was successful at eliciting responses in magnetoreceptive targets. Thus, this paper presents a system that provides the ability to conduct a parametric study of potential stimulation parameters and investigate the response thresholds of these parameters.

#### 2.1.1. Microscopy

Microscopy applications tend to impose strict size constraints on electromagnets. As also seen in Pashut et al. [10], care must be taken to ensure that the electromagnet does not interfere with the objectives or condenser of a microscope. For fluorescence microscopy, calcium imaging, voltage imaging, and patch clamping, a single coil was designed specifically to fit around a circular 35 mm diameter glass bottomed cell culture dish. During imaging, the electromagnet is either placed within a microscope compatible auxiliary incubation chamber or mounted underneath the stage directly below the sample, depending on the microscope in use.

**Figure 1:**
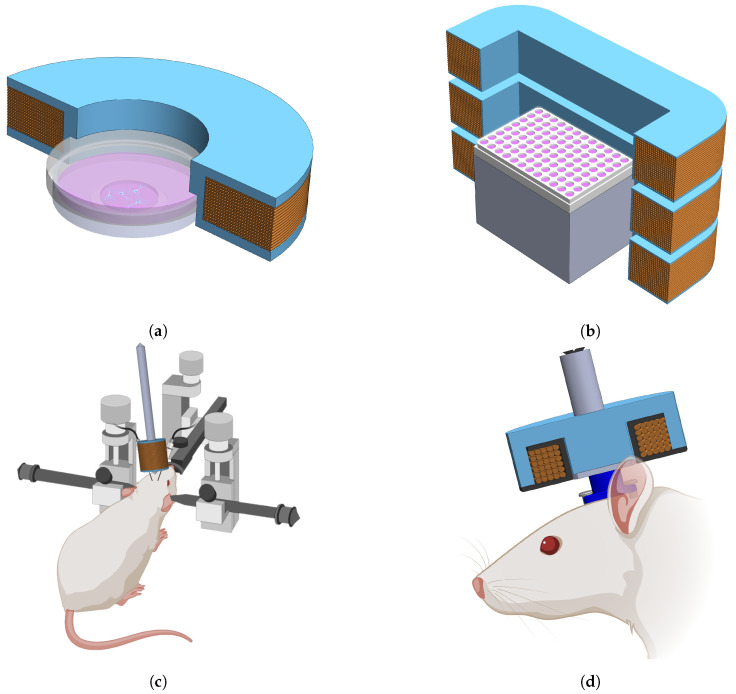
Stimulation applications are pictured for each of the four geometries under study, where (**a**,**b**,**d**) show half of the coils for improved visualization. (**a**) A coil designed to fit over top of a 35 mm culture dish which can be used for microscopy; (**b**) a set of three coils which can be stacked and used for stimulating samples on a multi-well plate with a relatively uniform field; (**c**) an application of an electromagnet for head-fixed electrophysiology; (**d**) a coil wound onto a bobbin and inserted into a pot core which could be used for freely behaving animal studies. Created in part using BioRender.com.

The incubation chamber restricts the maximum width and height of the coil holder to 85 mm and 15 mm, respectively. An assembled coil placed around a 35 mm culture dish is illustrated schematically in Figure 1a, where only half of the coil is shown for clarity. An advantage of this design, as will be shown in Section 2.2, is that the field in the target region at the center of the plate is relatively uniform, thereby providing the ability to reliably deliver consistent stimulus between experiments.

#### 2.1.2. Luminescence and Fluorescence Imaging

This application consists of measuring responses to magnetic stimuli in many cell preparations at the same time. A multi-well plate is used to test the effects of cell type, media preparation, control conditions, or genetic variants of a protein all within the same trial. This enables higher throughput for screening experiments, opening the door for mutagenesis studies aimed at improving stimulation responses. The application requires consistent stimulus delivery in each trial. To facilitate higher throughput screening, a three-coil electromagnet was designed based on the Merritt Coils outlined in [12,14,15] and used for stimulation of multi-well plates within a PerkinElmer In Vivo Imaging System (IVIS).

Using a multi-coil design aids in producing a uniform magnetic field within a volume along the central axis of the coil. Figure 1b shows an illustration of half of the three-coils with a 96-well plate placed at the central plane.

#### 2.1.3. In Vivo Electrophysiology

The potential neuromodulation effects of magnetic stimulation on rodents expressing the EPG protein or any other magnetoreceptive gene is considered in this application. An electromagnet design consisting of a coil wound around a ferromagnetic core is proposed. This coil was attached to an adjustable arm and positioned next to the head of a rat while recording neural signals.

This design is particularly useful when the application allows for less restrictions on the physical placement of the coil. A schematic of the experimental setup for this application is presented in Figure 1c, where the electromagnet is positioned between electrodes placed in the brain of an anesthetized, head-fixed rat. Typically, there is much more space to place the equipment and adjust it for proper alignment with the target in electrophysiology than in applications such as microscopy, making this a versatile solution.

#### 2.1.4. Freely Moving Animal

In addition to the aforementioned methods of investigating magnetosensitive pathways, it is also of great benefit to be able to study the effects of stimulation on the behavior of freely moving animals. Such studies could be performed in an operant conditioning box and designed to monitor reward seeking behavior, anxiety, stress, etc.

This application, however, presents a challenge for stimulus delivery. In the case of rodents, cages used for behavioral studies can vary from 200–500 mm in length and width, and could be as tall as 300 mm. While Merritt Coils can deliver uniform stimulus to a given volume, for delivering uniform fields to a large volume, the required power can exceed the capabilities of practical systems. Delivering a stimulus of up to ∼50 mT within a uniform field in a region the size of a rodent cage using Merritt Coils is therefore impractical.

Alternatively, a stimulus can be delivered locally using a fixed stimulation device attached to the subject. The attachment may consist of a head mounted fixture or a wearable jacket and would allow the animal to freely move about. Stimulus can then be applied to the localized area when desired. For this application, an electromagnet built into a pot core provides a good solution. Pot cores consist of a central rod around which a coil is wrapped with an additional metal shield which surrounds the coil. They are typically made of ferrite and are highly permeable to magnetic fields and therefore serve to increase magnetic flux density and focus the magnetic fields within the core region. A depiction of such a device is shown in Figure 1d, where it is attached to a custom head mounting fixture.

### 2.2. Numerical Modeling

Before fabricating and assembling the electromagnets and associated components, each design was numerically modeled and simulated using finite element analysis (FEA) to assess the performance of the design. Simulations were implemented using COMSOL Multiphysics and a standard linear solver was used to solve the electromagnetic field equations governing the underlying physics.

All simulations were performed with a constant excitation current of 15 Amperes to solve for the distribution of the magnetic flux density, **B**. The simulations are posed as magnetostatics problems, i.e., the electric field at the target location remained unaffected by the coil operation during stimulation. Visualization of the magnitude of **B**, |**B**|, served to both aid in optimization of the geometric parameters of the designs and provide a benchmark for experimental coil comparisons.

A summary of the parameters of the coils used in simulation is provided in Table 1, including the size of both the wire and device as well as the number of turns used. Additionally, values for the same parameters of the experimental devices are also listed, which are discussed in Section 2.3. In the case where the physically realized number of turns were less than initial estimations, the numerical models were updated to more accurately reflect the physical coils and facilitate more realistic comparison of coil simulations to experimental measurements.

#### 2.2.1. Air Core Model

**Figure 2:**
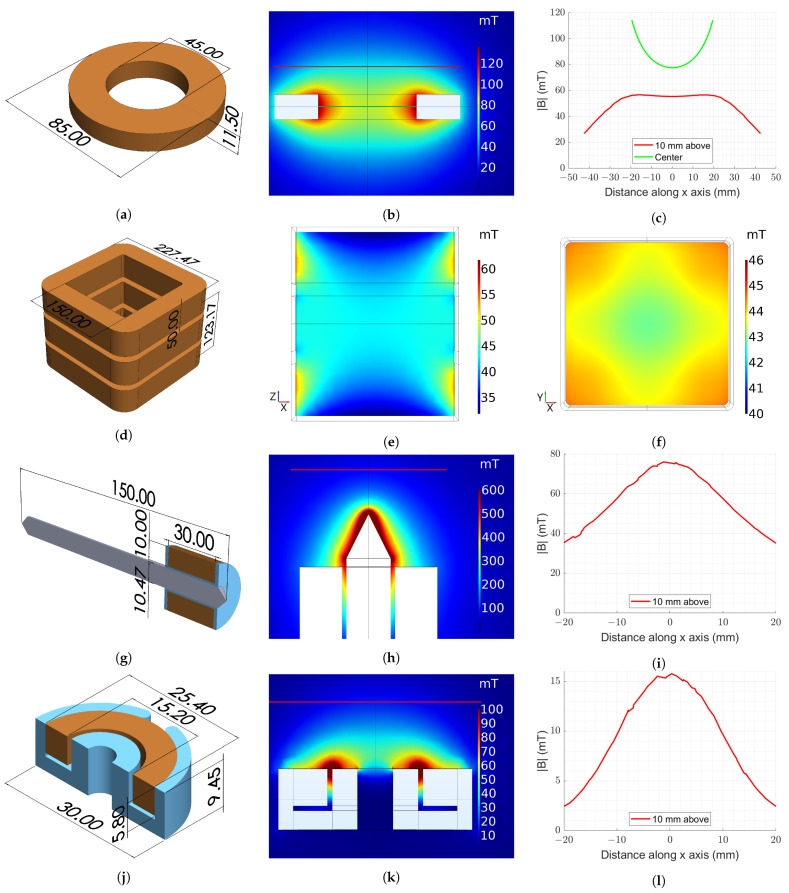
Electromagnet geometries and simulated |**B**| distributions. Dimensions for the (**a**) air core coil; (**d**) three-coil system; (**g**) ferromagnetic core coil; and (**j**) pot core coil. Simulated |**B**| distributions for the (**b**) air core coil; (**e**) three-coil system XZ plane; (**f**) three-coil system XY plane; (**h**) ferromagnetic core coil; and (**k**) pot core coil. The superimposed lines represent the location of the line scans (**c**) for the air core coil; (**i**) ferromagnetic core coil; and (**l**) pot core coil.

A single coil geometry was modeled to fully utilize the space available within the imaging incubation chamber for a Keyence BZ-X800E microscope. A coil with 264 turns, height of 11.5 mm, outer diameter of 85.0 mm, and inner diameter of 45.0 mm was considered. Figure 2a shows a schematic of the simulated coil and Figure 2b shows the simulation results of the |**B**| in the YZ plane. Figure 2c shows the simulated line scans along the lines depicted in Figure 2b, showing that stimulations greater than 50 mT are expected.

#### 2.2.2. Three-Coil System Model

A three-coil geometry was modeled to fit inside of an IVIS having an imaging chamber of dimensions 430 × 380 × 430 mm. Due to a 100 V supply voltage constraint and 15 Ampere supply current constraint, the total device resistance was constrained to 6.66 Ω. The coil was modeled with 12 gauge magnet wire, selected due to its low resistivity. The maximum length of the wire was determined based on the maximum resistance and wire resistivity.

Figure 2d shows a representation of the dimensions of the three-coil system. In addition, 150 mm was selected for the inner square side length since the multi-well plates are 130 mm wide. The total height of the system was chosen to be 123.17 mm, consistent with the ratio of side length to coil spacing presented in [14] for a three-coil Merritt Coil system,

(1) $$\frac{h}{d}=0.821116,$$

where *d* is the length of each side of the coils and *h* is the height. The height and outer width of each coil was selected to be 50 mm and 227.47 mm, respectively, resulting in a simulated coil thickness of 38.74 mm and 276 turns per coil.

A side view of the central XZ plane is shown in Figure 2e having a |**B**| of about 45 mT in the center. Figure 2f shows the central XY plane, which clearly demonstrates the uniformity of the field in a central circular region of about 80 mm in diameter.

In contrast to coils shown in [12,14,15] which use an ampere turn ratio of 0.512 for the center coil relative to the top and bottom coils, the system modeled utilizes coils of equal ampere turn ratios which adds more turns to the system. A central coil with ampere turn ratio 20:39 can be substituted should a volume of uniformity be required instead of a plane.

#### 2.2.3. Ferromagnetic Core Model

A ferromagnetic core of diameter 10.47 mm, length 150 mm, and relative magnetic permeability of 100,000 was modeled. The relative magnetic permeability value was modeled based on the property value reported in the material datasheet for the mu-metal rod described in Section 2.3.4. The tips at each end are tapered to a point as seen in Figure 2g. The coil geometry with an outer diameter of 31.97 mm wrapped along 30 mm of the length of the core. The coil was initially simulated with 372 turns; however, it was updated to have 308 turns to reflect the number of turns in the assembled coil.

Figure 2h shows that the field exceeds 600 mT at the very tip of the core. This flux then decays quickly along the coil axis, dropping to about 75 mT at a distance of 10 mm from the tip of the core, as seen in Figure 2i.

#### 2.2.4. Pot Core Model

Lastly, the pot core configuration for freely moving animals was simulated. For a pot core electromagnet to be head mounted on a rat, it must be small and light enough to allow maneuverability. A pot core geometry with an outer diameter of 30 mm and height of 9.45 mm was modeled along with the coil. A 28-turn coil was modeled to fit in the coil channel having depth and width of 6.5 mm and 6.05 mm, respectively. The core was simulated with a ferrite core having a relative magnetic permeability of 10,000, based on the property value reported in the datasheet for the pot core described in Section 2.3.5. Figure 2j shows the simulated pot core.

Since the device will be mounted on the moving animal, which is the target of stimulation, the magnetic field of interest is along the coil axis on the unshielded side of the pot core. The field distribution predicted by the numerical model is displayed in Figure 2k. While Figure 2k shows that there are strong fringing fields in close proximity to the coil, these fields decay quickly to yield uniform fields a few mm away. In practice, the coil holder and mounting device will cause the source to target distance to be a few mm. Figure 2l shows the magnetic field strength of the pot core at 10 mm from the device, indicating that a strength of ∼15.5 mT at the target is achievable. Even though the simulated target value falls below 50 mT, reaching such a stimulation strength in a compact device is thought to be sufficient for our experimental needs.

### 2.3. Magnet Assembly Implementation

#### 2.3.1. Double Wrapping Coils

**Figure 3:**
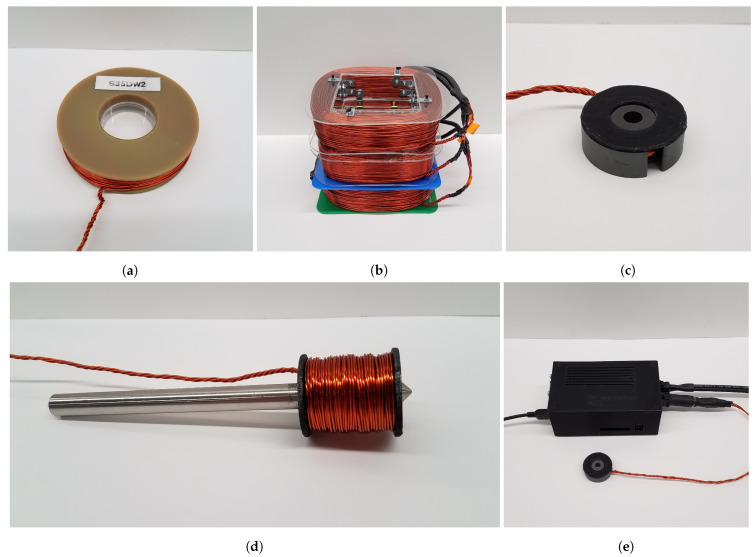
Assembled electromagnet devices. The air core coil in (**a**) is shown over top a 35 mm culture dish; (**b**) shows the stacked three-coil system, where each coil is connected in series; (**c**) pot core design shown with the unshielded, open side up; (**d**) ferromagnetic core coil; (**e**) The stimulation controller is pictured connected to a laptop with USB, the power supply which delivers the DC stimulation current, and the pot core coil.

All coils pictured in Figure 3 are double wrapped, as demonstrated in [8,12], to allow for a negative control. Wrapping a coil with two adjacent wires allows a user to reverse the direction of current in one wire relative to the other. This has the effect of canceling out the magnetic fields generated by the two opposing currents, thereby resulting in a net zero field. The stimulus can then be operated in either active or sham mode, which can help to provide a control for motion caused by the changing magnetic flux or temperature increase due to ohmic heating in the coils.

In practice, it is not possible to fully cancel out the magnetic field in the sham mode because this would require perfectly aligned wires with negligible width. Regardless, empirical measurements of the |**B**| of the sham conditions discussed herein are typically at least an order of magnitude smaller than the flux density delivered in the active mode.

Two types of magnet wire were used in the assembly of the coils, either the 20 gauge copper magnet wire MW0167 offered by TEMCo Industrial or the 12 gauge 12 HAPT-200 offered by MWS Wire Industries.

#### 2.3.2. Air Core

A coil holder was 3D printed from the high temperature plastic Digital ABS, heat treated, and then wrapped with 20 gauge magnet wire such that the channel was evenly filled, resulting in 280 total turns. Figure 3a shows the assembled air core coil placed over the top of a 35 mm culture dish. The assembled coil dimensions align closely with the simulated values that can be seen in Table 1.

#### 2.3.3. Three-Coil System

Three square coils were constructed to the same specifications used in the simulation model using 12 gauge magnet wire. The system is shown in Figure 3b with all three-coils together. Several methods were used in coil construction, with the initial method based on 3D printed parts. Due to the weight and size of the coils, the 3D printed parts were suboptimal in terms of strength and rigidity. Subsequent coil holders were assembled from acrylic components with metal hardware. While the top coil visible in Figure 3b is assembled with steel hardware, the central coil uses non-ferromagnetic brass hardware as described in [8].

The dimensions of the assembled coils of the three coil system, as seen in Table 1, match closely to simulated values. Some variation is present, as the size of the coils makes them difficult to assemble and and measure. Flexibility of the structural materials causes some bending in the coil holders due to the pressure applied by the tightly wound coil, which served to increase the overall height of the system by about 10 mm. In addition, improvements in the coil winding process allowed the final coil to be wound more tightly, thereby reducing the outer side length slightly. For these reasons, the use of an approximate outer side length of 230 mm is appropriate.

#### 2.3.4. Ferromagnetic Core

A 150 mm section of 0.412${}^{\prime \prime }$ EFI 50 round bar mu-metal from Ed Fagan was machined such that each tip came to a point as shown in Figure 2g. Two half bobbins were used when wrapping the coil around the ferromagnetic core so that the tightly wound wires would apply pressure on the half bobbins to maintain their position on the core. The total outer diameter of the coil is about 30.7 mm, with an inner coil diameter equal to 11.72 mm, accounting for the core diameter and 3D printed bobbin with Z-PETG. The metal core has a maximum relative magnetic permeability of 100,000 for DC operation, and 308 turns of 20 gauge magnet wire were used in the construction. Figure 3d shows the fully assembled ferromagnetic core electromagnet.

#### 2.3.5. Pot Core

For the pot core coil design, the ferrite pot core 0W43019UG from Magnetics Inc. was selected due to both its size and magnetic properties. The ferrite material selected has the highest relative permeability of pot cores produced by Magnetics Inc., measuring at 10,000 ± 30%. At 30 mm wide and 9.5 mm thick, this pot core is compact enough to be head mounted and large enough to fit a multi-turn 20 gauge wire coil.

To get the maximum number of windings into the pot core channel, the coil is tightly wound around a 3D printed bobbin printed with Zortrax Z-PETG material. Once wrapped, the bobbin is inserted into the pot core. A total of 28 turns of 20 gauge wire were fit into the channel. Figure 3c shows the assembled pot core device with the plastic bobbin inserted.

Attaching the pot core to the freely moving animal is also important for this application. To achieve secure attachment of the device while minimizing the distance between the coil and the stimulation target, a custom coil holder was designed to mount the pot core using the hole along the coil axis. This mount is itself attached to a permanent head mounted fixture that can be secured to the head of the animal. Alternatively, the device could also be attached to a wearable jacket to facilitate stimulation in other regions of an animal.

#### 2.3.6. Stimulation Controller

One of the goals of this work was to make the electromagnetic stimulation platform versatile and easy to use. Toward this goal, a stimulation controller was developed which allows the user to specify the stimulation protocol and switch between the active and sham conditions. Using automated stimulation protocols is highly advantageous as it increases the stimulation consistency between experiments.

A Python application was developed to set the stimulation protocol and allow the user to control the stimulation delivered by a custom hardware device shown in Figure 3e, enabling selection of the direction of current through the double wrapped coils from within the graphical interface. Additionally, stimulations can be triggered by an external signal, and an auxiliary stimulation signal can be connected to other devices.

## 3. Results and Discussion

It is crucial to validate simulated results with experimental measurements to fully characterize the core and coil parameters in an electromagnet stimulation system. For a quantitative comparison between simulation and experiment, we have utilized a TLE493D-A2B6 three-axis Gaussmeter along with an Aerotech AGS1000 programmable XYZ scanner to measure the distribution of **B** in the regions of interest as shown in Figure 2 produced by the different configurations of stimulation devices. All experimental scans were performed with step sizes of 0.5 mm.

All experimental measurements were performed with a 1 Ampere excitation current. At each position, five sensor measurements were averaged to generate the resulting |**B**|. To compare the experimental measurements with corresponding simulation results, magnetic flux density images were first aligned based on the maximum of their cross-correlation.

**Figure 4:**
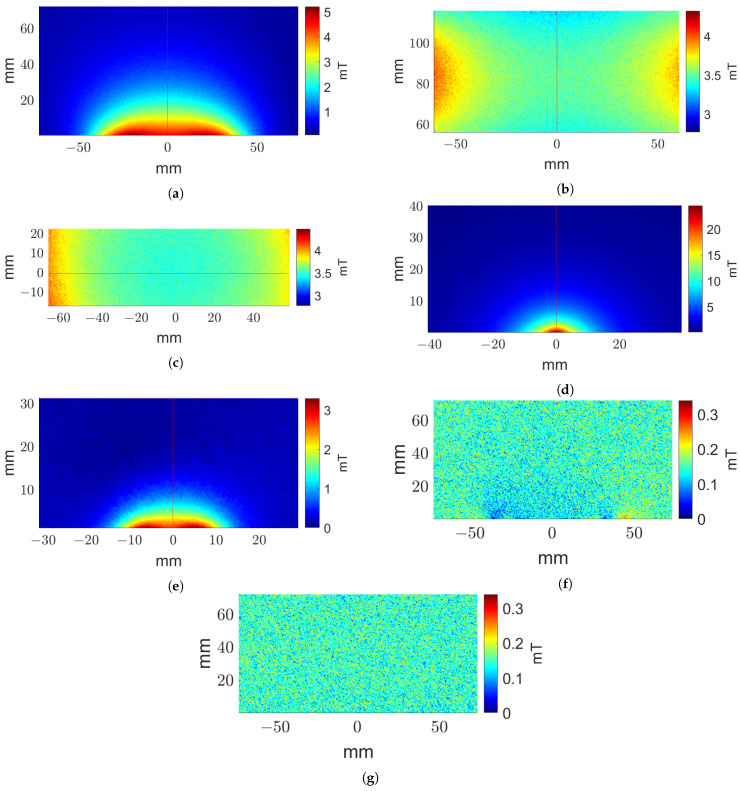
Experimental |**B**| distributions. (**a**) Air core coil active condition measurements along the central XZ plane. Three-coil system active condition measurements along the central (**b**) XZ and (**c**) XY planes; (**d**) ferromagnetic core coil active condition measurements along the central XZ plane; (**e**) pot core coil active condition measurements along the central XZ plane; (**f**) sham and (**g**) control condition measurements along the central XZ plane for the air core coil.

A qualitative comparison of the experimental and simulated field data are shown in Figure 4 for the air core, three-coil, ferromagnetic core, and pot core systems. The images represent the spatial distribution of |**B**|. Results for the air core coil, shown in Figure 4a, indicate that a maximum |**B**| of 5.20 mT is recorded just above the surface of the coil. For the three-coil system, the first region of interest consists of the central XZ plane passing through the coils. A region of 60 mm by 120 mm centered at the center of the coil was scanned in the XZ plane, as shown in Figure 4b. In addition, measurements along the central XY plane were performed along a 40 mm by 120 mm region approximately centered on the coil axis, as shown in Figure 4c. In Figure 4d, we see the measured distribution of |**B**| for the ferromagnetic core coil, reaching a maximum of 24.55 mT. It is not surprising that, of the four geometries, this design produces the highest |**B**| at the target location due to the effect of the ferromagnetic core. The pot core measurements are presented in Figure 4e, where the |**B**| achieves a maximum of 3.29 mT.

In addition to measuring the |**B**| during the active condition, similar measurements were taken in the sham configuration as well as with no stimulation current. The air core sham results are seen in Figure 4f, while the results for the air core no stimulation current control are shown in Figure 4g. Low amplitude fringing fields are observed near the coil at the bottom of the image in the case of the sham condition. Otherwise, the sham condition performed similarly to the no current case. Similar results were observed in the case of other geometries.

**Figure 5:**
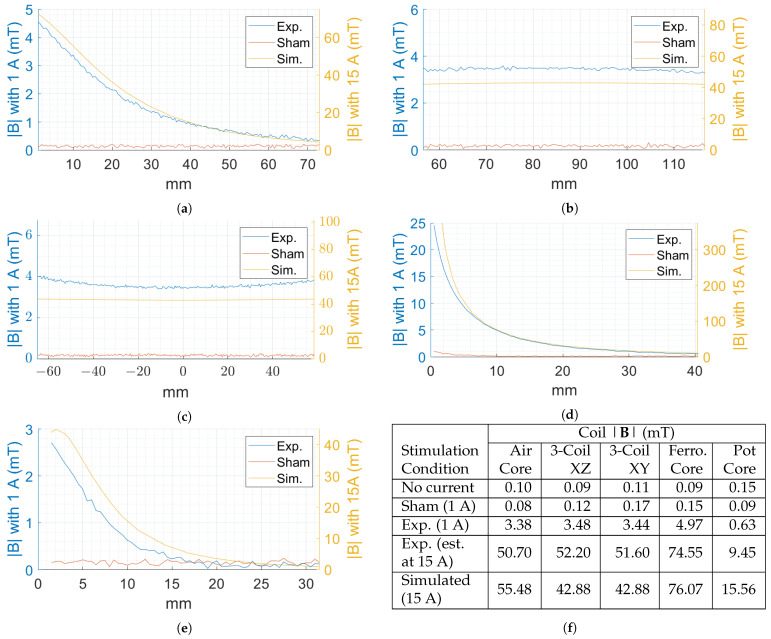
Line scans showing the experimental, sham, and simulated |**B**| along the red line depicted in the corresponding distribution images of Figure 4 for the (**a**) air core coil; (**b**) three-axis XZ; (**c**) three-axis XY, (**d**) ferromagnetic core coil; and (**e**) pot core coil scans. Experimental measurements were performed under a 1 A stimulation, simulated measurements were performed with 15 A, and the left and right axes of the graphs show the relationship between the |**B**| for a 1 A and 15 A stimulation, respectively; (**f**) summary of the |B| for the control, sham, experimental, and simulated conditions. Additionally, estimations of the |**B**| for each coil under a 15 A stimulation are included by linearly scaling the results from a 1 A stimulation by a factor of 15. Measurements are from a distance 10 mm from the coil along the central axis for the air, ferromagnetic, and pot core coils and at the center for the three-coil system.

A quantitative comparison of the predicted and measured fields for the no current, sham, experimental, and simulated stimulations for each coil design is shown in Figure 5 and summarized quantitatively in Figure 5f. The values listed in Figure 5f for the cases of air, ferromagnetic, and pot core coils are measured at a distance of 10 mm from the coil along the coil axis, while measurements for the three-coil system are taken at the center of the XZ and XY planes.

With regard to the uniformity of the stimulus delivered by the three-coil system, Figure 5b shows that the flux density along the three-coil system’s XZ axis drops on average only 2.99% in strength at the extrema of the line scan compared to the center. In the XY plane, the |**B**| increases on average 5.98% at ±40 mm from the center, whereas, at the extrema of the line scan, it increases on average by 14.33% compared to the center.

It is worth noting that the ferromagnetic core does retain a low level of magnetization. However, the rapid decay in |**B**| means the magnetization has little effect at a distance of 10 mm from the tip.

Performance of the pot core coil showed that it did not reach the stimulation strength of the simulated target value. It is possible that the wide tolerance of the magnetic permeability of the pot core, ±30%, played some role in contributing to the reduced strength. Regardless, achieving a stimulation strength of 9.45 mT at the target distance is thought be a successful implementation of such a compact stimulation device. Future work will aim to increase the strength of the device by reducing the target distance to less than 10 mm and using a smaller diameter magnet wire to increase the number of turns. In the case of the latter method for increasing stimulation strength, careful consideration must be given to the trade-off between added performance in strength and the decreased thermal performance, which is a consequence of using smaller wire.

Despite the use of DC stimulation in our study, induction of a transient electric field is inevitable during the powering on and powering off of the stimulation. To characterize the induced electric field at the target locations during these periods, electrical characteristics of the coils were measured and the induced electric field was estimated. Table 2 shows the resistance and inductance used to determine the rise and fall times according to the relationship

(2) $$t_{r}=t_{f}=\frac{L}{R}\ln9,$$

where $t_{r}$ is rise time, $t_{l}$ is fall time, *L* is inductance, and *R* is resistance. Equation (2) can be derived from the equations governing a step response in an RL circuit. The rise or fall times indicate the time required for the stimulation to ramp up from 10% strength to 90% strength or vice versa. An estimation of the induced transient electric field at the target locations is also shown in Table 2, calculated using Faraday’s Law of induction over the transition from 10% to 90% stimulation strength.

Thermal imaging was also performed with a FLIR One thermal infrared camera. Safe operation of stimulation coils requires identification of maximum operating times for each coil geometry to stay below 75 °C. Such analysis is important to carefully design experiments that will allow the coils to stay within the defined temperature limits. Currents ranging from 1 to 15 Amperes at 1 Ampere intervals were applied to stimulate each coil while sampling coil temperature at one sample per second until the coil temperature reached 75 °C.

**Figure 6:**
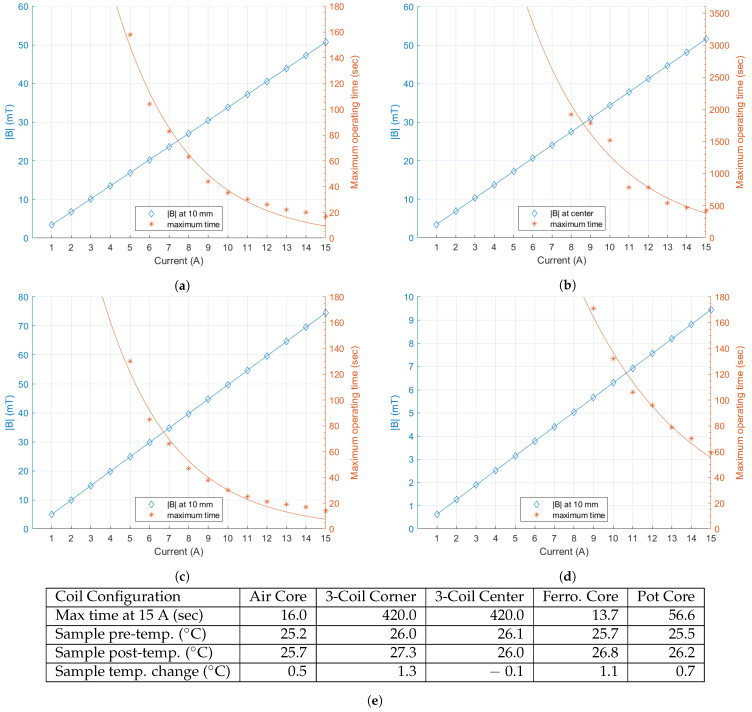
|**B**| at 10 mm from the coil or the center for the three-coil system, shown on the left axis for a range of 1 to 15 Amperes for the (**a**) air core coil; (**b**) three-coil system; (**c**) ferromagnetic core coil; and (**d**) pot core coil. In addition, the maximum operating time along the right axis for the same conditions is shown. Operating times are shown only up to one hour for the three-coil system and three minutes for the remaining geometries; (**e**) summary of the device operating times and sample temperatures for each coil design.

Results plotted in Figure 6 can be used to determine both the maximum excitation current and stimulation time based on the desired stimulus strength. For each of the four geometries, composite plots of maximum flux density magnitude and maximum operating time are shown for increasing stimulation current values ranging from 1 to 15 Amperes. The blue *y*-axis on the left shows the flux density magnitude in mT and the orange *y*-axis on the right shows the maximum operating times for the given stimulation current. Stimulation times are cutoff after one hour for the three-coil system and three minutes for the remaining geometries. An exponential best fit line for the maximum stimulation times is also shown in each graph of Figure 6.

Temperatures were also measured at sample target locations for each coil configuration with a 15 Ampere stimulation current. Target locations for temperature measurements were the same locations as the measurements from Figure 5f. Additionally, temperature measurements were taken at the corner wells of a 96-well plate placed in the three-coil system. For the three-coil system, temperature was observed after application of a five minute stimulation. The air core, ferromagnetic core, and pot core coils were stimulated for the duration of their respective maximum operating times indicated in Figure 6.

With the air core coil, sample temperature was seen to rise by 0.5 °C, whereas the ferromagnetic core sample temperature increased by 1.1 °C and the pot core sample temperature increased by 0.7 °C throughout the stimulations. The temperature of the corner well samples in the three-coil system showed an increase of 1.3 °C after five minutes, while the temperature at the center remained essentially unchanged, decreasing by 0.1 °C.

## 4. Conclusions

This study presented a magnetogenetics stimulation platform that supports four electromagnet stimulation coil designs and a controller for selecting stimulation conditions. The various coil geometries were chosen so that at least one of the designs satisfied the needs of magnetogenetics experiments including microscopy, in vivo electrophysiology, freely moving behavioral experiments, and some fluorescence and luminescence imaging setups.

Regarding the use of ferromagnetic materials in stimulation coils, the added benefit of increased stimulation strength for an otherwise similar coil without a ferromagnetic core must be weighed against the necessity to account for the residual magnetization of the material. Negative effects can be mitigated by either demagnetizing the core between stimulations or placing the coil at a distance such that the residual field of the core does not interfere with the experiment design.

While sham conditions are required to ensure proper experimental controls, it is important to understand their limitations as they apply to a given experimental protocol and stimulation conditions. The sham conditions all demonstrated at least an order of magnitude reduction in |**B**|; however, some residual magnetic flux is unavoidable. To properly incorporate sham conditions into an experiment, it would be best to know the minimum stimulation threshold necessary to produce a meaningful target response. With this knowledge, stimulations can be performed such that active conditions provide suprathreshold stimulation while sham conditions provide only subthreshold stimulation.

Accounting for the effects of temperature change is important when studying pathways with thermal sensitivity. Our designs showed minimal temperature increases (0.5–1.3 °C) at sample locations under maximum field strength conditions in all cases studied.

The versatility of various magnet designs presented allows for multiple choices of electromagnets based on the size constraints of the application. The analysis of the |**B**| distributions is important for selecting an appropriate electromagnet system to achieve the proper strength of the applied stimulus at the target location to successfully elicit a response. Additionally, our analysis of the sham stimulus strength is important in designing experiments with a negative control which can help eliminate the role of confounding variables on observed effects.

Studies presented here also provide a useful tool for selecting experimental design parameters for magnetogenetics experiments. For example, knowing that the three-coil system has a field distribution that varies less than 6% over a range of 40 mm from the central axis of the coils means that the sample placement should be restricted to this range in order to maintain a high degree of stimulation uniformity. In addition to limiting the effects of temperature on experimental observations, thermal analysis also allowed for determination of safe operating limits for the coils. Figure 6 can be used to find the operating limits, in terms of time and current, for a desired |**B**| with each coil geometry. Lastly, the use of a custom stimulation controller allows for easily configurable stimulation patterns, in either the sham or experimental modes, which improves repeatability.

## Figures and Tables

**Table 1:** Parameters of the simulated and experimental coils including the type of wire, materials, size, and number of turns for each.

Coil	Simulated or Experimental	Wire	Materials	O.D. or Side Length (mm)	I.D. or Side Length (mm)	Height (mm)	Number of Turns
Air Core	Simulated	20 AWG	Air	85.00	45.00	11.50	264
	Experimental	20 AWG	Digital ABS	∼84.0	45.00	11.50	280
3-Coil System (Single coil)	Simulated	12 AWG	Air	227.47	150.00	50.00	276
	Experimental	12 AWG	PLA, Acrylic	∼230.0	150.00	∼5.0	276
3-Coil System (3 coils)	Simulated	12 AWG	Air	227.47	150.00	17.32	3 × 276
	Experimental	12 AWG	PLA, Acrylic	∼230.0	150.00	∼18.4	3 × 276
Ferromagnetic Core	Simulated	20 AWG	mu-metal	31.97	11.97	30.00	308
	Experimental	20 AWG	mu-metal, Z-PETG	∼30.7	11.72	30.00	308
Pot Core	Simulated	20 AWG	Ferrite	25.40	15.20	5.80	28
	Experimental	20 AWG	Ferrite & Z-PETG	25.40	15.20	5.80	28

**Table 2:** Electrical parameters of the stimulation coils. Rise/Fall time indicates the time required to transition between 10% to 90%, or 90% to 10%, stimulation strength when the stimulation switches on or off. The transient E-field is estimated by Faraday’s Law of induction during these periods, where the change in flux is estimated based on the predicted experimental 15 A |**B**| reported in Figure 5f.

Coil	Resistance	Inductance	Rise/Fall Time	Transient E-Field
Air Core	2.3 Ω	5.2 mH	5 msec	8.1 V/m
3-Coil System	3.3 Ω	95.1 mH	63.3 msec	659.7 mV/m
Ferromagnetic Core	1.0 Ω	3.7 mH	8.1 msec	7.4 V/m
Pot Core	0.3 Ω	62.3 μH	456.3 μsec	16.6 V/m

## Data Availability

Not applicable.

## Abbreviations

The following abbreviations are used in this manuscript:

EPG	Electromagnetic Perceptive Gene
IVIS	In Vivo Imaging System
FEA	Finite Element Analysis
